# Ctr9, a Key Component of the Paf1 Complex, Affects Proliferation and Terminal Differentiation in the Developing *Drosophila* Nervous System

**DOI:** 10.1534/g3.116.034231

**Published:** 2016-08-11

**Authors:** Shahrzad Bahrampour, Stefan Thor

**Affiliations:** Department of Clinical and Experimental Medicine, Linkoping University, SE-58185, Sweden

**Keywords:** neuroblast, lineage tree, cell cycle, epigenetics, terminal differentiation, FlyBook

## Abstract

The Paf1 protein complex (Paf1C) is increasingly recognized as a highly conserved and broadly utilized regulator of a variety of transcriptional processes. These include the promotion of H3K4 and H3K36 trimethylation, H2BK123 ubiquitination, RNA Pol II transcriptional termination, and also RNA-mediated gene silencing. Paf1C contains five canonical protein components, including Paf1 and Ctr9, which are critical for overall complex integrity, as well as Rtf1, Leo1, and Cdc73/Parafibromin(Hrpt2)/Hyrax. In spite of a growing appreciation for the importance of Paf1C from yeast and mammalian studies, there has only been limited work in *Drosophila*. Here, we provide the first detailed phenotypic study of *Ctr9* function in *Drosophila*. We found that *Ctr9* mutants die at late embryogenesis or early larval life, but can be partly rescued by nervous system reexpression of *Ctr9*. We observed a number of phenotypes in *Ctr9* mutants, including increased neuroblast numbers, increased nervous system proliferation, as well as downregulation of many neuropeptide genes. Analysis of cell cycle and regulatory gene expression revealed upregulation of the E2f1 cell cycle factor, as well as changes in Antennapedia and Grainy head expression. We also found reduction of H3K4me3 modification in the embryonic nervous system. Genome-wide transcriptome analysis points to additional downstream genes that may underlie these Ctr9 phenotypes, revealing gene expression changes in Notch pathway target genes, cell cycle genes, and neuropeptide genes. In addition, we find significant effects on the gene expression of metabolic genes. These findings reveal that *Ctr9* is an essential gene that is necessary at multiple stages of nervous system development, and provides a starting point for future studies of the Paf1C in *Drosophila*.

During embryonic development, there is dynamic interplay between the primary level of transcriptional control, as chiefly executed by transcription factors and cofactors, and the epigenetic machinery (Cantone and Fisher 2013; Leeb and Wutz 2012). A number of epigenetic complexes have been identified, some with highly restricted enzymatic roles, such as the trimethylation of H3K27 by Polycomb Repressor Complex 2 (Muller and Verrijzer 2009). In contrast, other complexes have more pleiotropic roles, apparently affecting several epigenetic and transcriptional processes; the Polymerase-Associated Factor 1 (Paf1) complex (Paf1C) belongs to this latter category (Jaehning 2010; Tomson and Arndt 2013).

Paf1 was first identified in *Saccharomyces cerevisiae* by its interaction with RNA polymerase II (RNA pol II) (Shi *et al.* 1996). Related studies further identified the Cell Division Cycle 73 protein (Cdc73; denoted Parafibromin/Hrpt2 in mammals and Hyrax in *Drosophila*) as copurifying with Paf1 and RNA pol II (Shi *et al.* 1997). Subsequently, three additional proteins were identified as being part of the yeast: Paf1C:Ctr9, Leo, and Rtf1 (Mueller and Jaehning 2002; Squazzo *et al.* 2002) (Figure 1M). Ctr9 (Cln Three Requiring 9) had also been identified genetically based upon its role in controlling the yeast cell cycle (Di Como *et al.* 1995; Foreman and Davis 1996). In yeast, Paf1C genes are not essential during optimal conditions, but affect the expression levels of numerous genes. In contrast, in metazoans, several members of the complex are essential, and have demonstrated effects on cell cycle and DNA repair and development (Jaehning 2010; Newey *et al.* 2009; Tomson and Arndt 2013). Several studies have furthermore identified links between Paf1C and Notch or Wnt signaling (Mosimann *et al.* 2006; Mosimann *et al.* 2009), as well as between Paf1C members and cancer (Dey *et al.* 2011; Hanks *et al.* 2014; Muntean *et al.* 2010; Newey *et al.* 2009; Takahashi *et al.* 2011; Zeng and Xu 2015). At the molecular level, Paf1C controls a number of transcriptional and epigenetic processes. These involve, but are not limited to promotion of H3K4 and H3K36 trimethylation, recruitment and activity of the Rad6-Bre1 complex (which ubiquitinates H2BK123), recruitment of the Chd1 chromatin remodeling factor, and proper RNA Pol II transcriptional termination (Jaehning 2010; Tomson and Arndt 2013). More recent studies have expanded these pleiotropic transcriptional functions of Paf1C to include roles in histone turnover and chromatin states, RNA pol II phosphorylation, pausing, and release, as well as RNA-mediated epigenetic gene silencing (Chen *et al.* 2015; Kowalik *et al.* 2015; Sadeghi *et al.* 2015; Yu *et al.* 2015).

**Figure 1:**
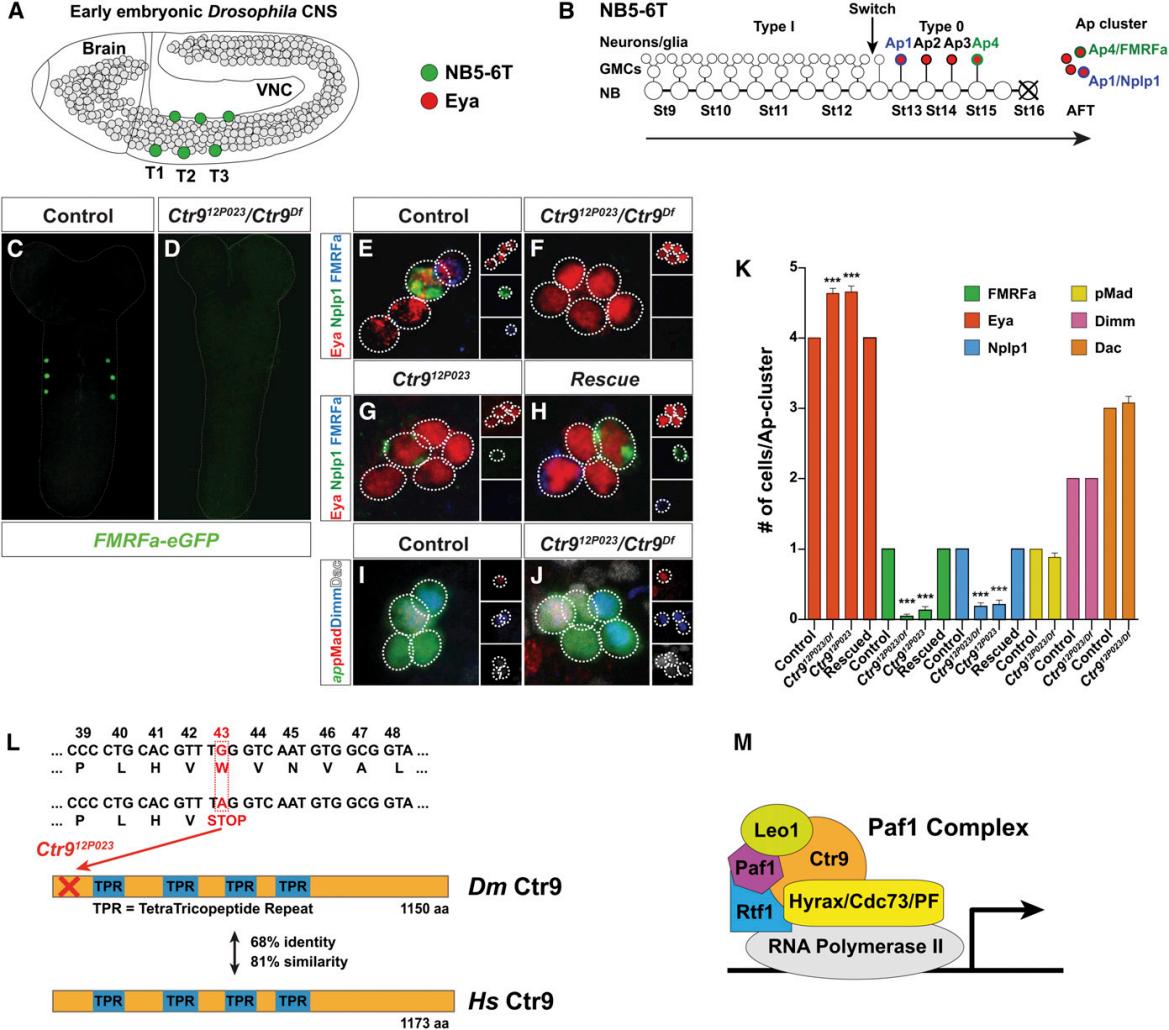
A genetic screen for FMRFa expression identifies *Ctr9*. (A) Cartoon of the embryonic *Drosophila* embryo, depicting neuroblasts (NBs) in the CNS (gray circles). NB5-6 is generated along the entire CNS, but generates a specialized lineage in the three thoracic (T) segments (green circles). (B) Cartoon of the NB5-6T lineage. This NB undergoes nine rounds of Type I proliferation, budding off daughters that divide to generate two neurons per glia. Subsequently, it switches to Type 0 division, generating daughter cells that directly differentiate. Among the latter group of cells are the Ap neurons, which are born at the end of the lineage, with the FMRFa neuron born last. The NB exits the cell cycle at St15 and undergoes apoptosis (marked by X) at St16. (C) In control, *FMRFa-eGFP* labels the thoracic FMRFa Ap neurons. (D) In *Ctr9* mutants, there is near complete loss of eGFP expression. The stain is eGFP fluorescence, no antibody stain. Magnification is 200x. (E and F) Close-up of the four Ap neurons, identified by expression of Eya, FMRFa, and Nplp1, in control and *Ctr9* mutants. In control, all four Ap neurons express Eya, while one cell expresses FMRFa and one Nplp1. In *Ctr9* mutants, one extra Ap neuron is often observed, while FMRFa and Nplp1 expression is frequently lost. (G) Homozygous *Ctr9^12P023^* mutants show similar phenotype as hemizygous mutants. (H) Both cell number and neuropeptide expression can be rescued by expressing *UAS-Ctr9* from the pan-neural *Gal4* driver *insc-Gal4*. (I and J) Expression of *ap^Gal4^*, Dimm, Dac, and pMad in control and *Ctr9* hemizygous mutants. Control display four *ap^Gal4^* neurons, out of which one expresses pMad, two Dimm, and one Dac. *Ctr9* mutants display one extra *ap^Gal4^* cell, which also expresses Dac and Dimm, while pMad appears unaffected. (K) Quantification of the observed phenotypes (*** *P* ≤ 0.001; Student’s two-tailed *t*-test ± SD; *n* ≥ 6 clusters). (L) (top) Sequencing of *Ctr9^12P023^* revealed a nonsense mutation early in the open-reading frame, converting a Trp to Stop; (bottom) the *Drosophila* Ctr9 protein shows a high degree of conservation to human Ctr9. (M) Ctr9 is part of the Paf1 complex, together with the other core components Leo1, Paf1, Rtf1, and Cdc73/Parafibromin/Hyrax, all of which are highly conserved throughout metazoans. Genotypes: (C) *FMRFa-eGFP*; (D) *FMRFa-eGFP*, *UAS-myr-mRFP*, *Ctr9^12P023^/Ctr9^Df^ = Df(3L)BSC250*; (E) *OregonR*; (F) *Ctr9^12P023^/Ctr9^Df^ = Df(3L)BSC250*; (G) *Ctr9^12P023^*; (H) *Ctr9^12P023^*, *UAS-Ctr9/insc-Gal4*.

In contrast to the extensive studies of Paf1C in both yeast and mammals, studies in *Drosophila* are more limited. *Cdc73* (*hyrax* in *Drosophila*) is an important mediator of both Wnt and Hedgehog signaling (Mosimann *et al.* 2006; Mosimann *et al.* 2009). *Rtf1* is an essential gene (Tenney *et al.* 2006), important for H3K4 methylation (Adelman *et al.* 2006; Tenney *et al.* 2006). *Paf1* (*antimeros* in *Drosophila*) is important for H3K4 methylation (Adelman *et al.* 2006), and is described as an essential gene (Spradling *et al.* 1999). *Leo1* (*another transcription unit* in *Drosophila*) has not been extensively studied, but is also known as an essential gene (Spradling *et al.* 1999). Finally, *Ctr9* (denoted *CG2469* by Flybase) has not been previously studied.

In a recent forward mutagenesis screen for genes affecting expression of a neuropeptide-GFP transgene (*FMRFa-eGFP*), we identified a mutant allele in *Ctr9* (Bivik *et al.* 2015). Here, we analyzed *Ctr9* mutants in detail. We found that *Ctr9* is an essential gene, with lethality during late embryogenesis and early larval life. Lethality can be partly suppressed into early larval stages by *Gal4/UAS*-mediated rescue in the developing nervous system. We also found that *Ctr9* is involved in proliferation control in the developing central nervous system (CNS), with mutants displaying increased proliferation both of neuroblasts (NBs) and their daughter cells. This phenotype may, in part, be explained by the elevated number of NBs that we observe in *Ctr9* mutants, but also by the altered expression of the cell cycle factor E2f1, as well as two transcription factors involved in proliferation control: Grainy head and Antennapedia. In line with the known role of Paf1C in specific histone modifications, in *Ctr9* mutants we found reduction of H3K4me3 modification in developing NBs. In addition to downregulation of the FMRFa neuropeptide, we found that *Ctr9* controls terminal differentiation of other neurons, evidenced by loss/reduction of expression of several other neuropeptide genes. Finally, genome-wide transcriptome analysis of *Ctr9* mutants revealed changes in several Notch pathway target genes, as well as cell cycle and neuropeptide genes. In addition, we observed expression changes in genes involved in metabolism. This provides the first functional study of the highly conserved Paf1C member *Ctr9* in *Drosophila*, demonstrating the fundamental role it plays during development, and provides a platform for future studies on this essential epigenetic complex.

## Materials and Methods

### Fly stocks

The following fly stocks were used: *lbe(K)-EGFP* (Ulvklo *et al.* 2012); *E(spl)m8-EGFP* (Castro *et al.* 2005) (provided by J. Posakony); *UAS-nls-myc-EGFP*, referred to as *UAS-nmEGFP* (Allan *et al.* 2003); *lbe(K)-lacZ* (Baumgardt *et al.* 2014); *Ctr9^Df^* = *Df(3L)BSC250* (Bloomington stock #23150); *hyx^2^* and *hyx^3^* (Mosimann *et al.* 2006) (provided by K. Basler); and *eg^Mz360^*, referred to as *eg^Gal4^* (BL#8758).

*UAS-Ctr9* transgenic strain was generated by inserting *Ctr9* cDNA clone LD24034 (from *Drosophila* Genomics Resource Center) into the pUASattB vector, and generating transgenes by PhiC31 transgenic integration (Bischof *et al.* 2007) (BestGene Inc., Chino Hills, CA), into cytological site *28E* on chromosome 2 (BL#9723).

*FMRFa-eGFP*, *UAS-myr-mRFP*, and *Ctr9^12P023^* (BL#59389) (Bivik *et al.* 2015) stock were outcrossed to *w^1118^* to remove both of the transgenic inserts, and other than Figure 1, C and D, a “clean” *Ctr9^12P023^* stock was used in this study.

Mutants were maintained over *GFP*- or *YFP*-marked balancer chromosomes; wild type, *w^1118^*, or *OregonR* was used. Staging of embryos was performed according to Campos-Ortega and Hartenstein (1985).

### Immunohistochemistry

Immunohistochemistry was performed as previously described (Baumgardt *et al.* 2009). Primary antibodies were: guinea pig α-Dap (1:1000), rat α-E2f (1:100) (Baumgardt *et al.* 2014); guinea pig α-Dpn (1:1000), rat α-Dpn (1:500) (Ulvklo *et al.* 2012); rabbit α-phospho-histone H3-Ser10 (PH3) (1:1000) (Upstate/EMD Millipore, Billerica, MA); rat α-PH3 (1:1000; Abcam, Cambridge, UK); rabbit α-β-Gal (1:5000; ICN-Cappel, Aurora, OH); chicken α-GFP (1:2000; Molecular Probes, Eugene, OR); chicken α-proNplp1 (1:1000) and rabbit α-proFMRFa (1:1000) (Baumgardt *et al.* 2007); rat α-Grh (1:1000) (Baumgardt *et al.*, 2009); rabbit α-Cas (1:2000) (Kambadur *et al.* 1998) (provided by W. Odenwald); rat mAb α-GsbN (1:10) (Buenzow and Holmgren 1995) (provided by R. Holmgren); mouse mAb α-Dap (1:500), mAb α-Antp (1:10), mAb α-Pros MR1A (1:10), and mAb α-Eya 10H6 (1:200) (Developmental Studies Hybridoma Bank, Iowa City, IA); rabbit α-CycE (1:500) (Santa Cruz Biotechnology, Santa Cruz, CA; and rabbit α-H3K4 trimethylation (1:1000; Abcam, AB8580).

Antibodies to Capa, CCAP, Crz, and Lk propeptides were generated by injecting animals with synthetic peptides (synthesized at Innovagen, Lund, Sweden) and conjugated to Keyhole limpet hemocyanin (KLH). proCapa (CKRSVDAKSFADISKGQKELN) was injected into two rabbits, proCCAP (CKQKMLQNEKEMQQLEERESK) was injected into two rabbits, proCrz (CLEELSAAGGASAEPNVFGKH) was injected into four hens, and proLk (CQRFHSWGGKRSPEPPILPDY) was injected into two rabbits. The N-terminal cysteine was added to allow for affinity purification. Sera from animals showing specific staining were purified using UltraLinkTM Iodoacetyl columns (PIERCE) and eluted at pH 7.0 using ActiSep (Sterogene). Peptide conjugation, animal sera production, and affinity purification was conducted by Agrisera (Umea, Sweden). Antibodies were used at anti-proCapa (1:500), anti-proCCAP (1:500), anti-proCrz (1:1000), and anti-proLk (1:500).

### Confocal imaging, intensity measurements, and image analysis

Fluorescent images were captured on Zeiss LSM700 confocal microscopes and confocal stacks were visualized utilizing LSM software. Intensity measurement was done using ImageJ software, and control was always stained on the same slide and scanned using the same settings as for mutants. Statistical analysis was done using GraphPad Prism software. Adobe Photoshop and Adobe Illustrator was used to compile figures and graphs.

### Sequencing of the Ctr9^12P023^ allele

DNA of homozygous *Ctr9* mutants was obtained from embryos at late stages, utilizing DNaesy Blood and Tissue Kit 5 (Qiagen, Hilden, Germany). A pair of primers were used to amplify a genomic 3770-bp segment, containing the eight exons of *Ctr9*, (forward primer: TGCATCAGCCGAGTAGAGA; reverse primer: AATCCCGGTTTGCCAGGTTT). Cloning of the PCR product was done by TOPO-TA Cloning Kit (Invitrogen) and sequenced (GATC Biotech, Konstanz, Germany), utilizing M13 forward and reverse primers.

### RNA sequencing and analysis

RNA was extracted from frozen collections of homozygous *Ctr9* mutants and wild-type embryos (50 mg), at late air-filled trachea (AFT) stage, using RNeasy Mini Kit (50) (Qiagen). Transcriptome sequencing of extracted RNA from samples was performed by GeneWhiz (South Plainfiled, NJ) on one lane of the HiSeq2500 with a 1 × 50 bp single-read sequencing configuration, which provided an average of 38 million reads per sample . RNA expression profiling and fold change comparison was performed using DNAStar 12.2.0.80, SeqMan NextGen software, performed in reads per kilobase of axon per million mapped sequence reads (RPMK). DNASTAR navigator 12.2.0.80 was used to search for RNA expression profiling, and gene ontology (GO) analysis were carried out with the web-based software GeneCodis (Carmona-Saez *et al.* 2007; Nogales-Cadenas *et al.* 2009; Tabas-Madrid *et al.* 2012).

### Data availability

The authors state that all data necessary for confirming the conclusions presented in the article are represented fully within the article. Gene expression data is available upon request.

## Results

### Forward genetic screen for FMRFa-eGFP expression identifies Ctr9

To identify genes involved in nervous system development, we recently conducted a forward genetic screen focusing on the lineage and neuronal progeny of thoracic neuroblast 5-6 (NB5-6T) in the *Drosophila* ventral nerve cord (VNC) (Figure 1, A and B). We chose the NB5-6T lineage to identify novel genes required for lineage progression and terminal fate differentiation for numerous reasons, which together make it a very useful model. First, a unique feature of this rather large lineage is the highly selective expression of the neuropeptide FMRFa in the last neuron formed in the NB5-6T lineage, and hence in only six cells of the embryonic VNC (Figure 1, B and C). Second, this lineage has a programmed switch in its proliferation mode during lineage progression, switching from a Type I mode (generating daughters that divide once) to Type 0 mode (generating daughters that differentiate directly into postmitotic cells) for the last five divisions (Figure 1B) (Baumgardt *et al.* 2014; Baumgardt *et al.* 2009). The last four cells born in the lineage are the Apterous neurons (Ap cluster), and the last-born cell is the Ap4/FMRFa neuron. Hence, expression of *FMRFa-eGFP* in the last-born cell in the NB5-6T lineage (*i.e.*, in a Type 0 daughter cell) not only identifies novel mutants that affect cell fate specification and differentiation, but also those that regulate proliferation control.

Using *FMRFa-eGFP* expression as marker, an EMS forward genetic screen for GFP expression in the *Drosophila* VNC was previously conducted, involving some 10,000 mutant lines (Bivik *et al.* 2015). One complementation group identified in the screen, displaying loss of *FMRFa-eGFP*, was mapped to the *CG2469* gene (Figure 1, C and D) (Bivik *et al.* 2015). *CG2469* encodes the single *Drosophila* ortholog of the *Ctr9* gene; a member of the Paf1 complex (Paf1C; Figure 1M), and we will refer to *CG2469* as *Ctr9*. The mouse and human genomes also contain a single *Ctr9* gene, to which *Drosophila* shows an overall amino acid sequence identity of 68%, and a similarity of 81% (Figure 1L). In addition to a loss of *FMRF-eGFP* and proFMRFa neuropeptide expression, *Ctr9* mutants often displayed a loss of the Nplp1 neuropeptide, normally expressed by the Ap1 neuron (Figure 1, E, F, and K). In contrast to loss of these terminal markers, there was often an extra Ap neuron in the cluster, and no loss of Ap neuron cell fate determinants, such as Dimm, Dac, and phosphorylated-Mad (Allan *et al.* 2005; Allan *et al.* 2003; Hewes *et al.* 2003; Miguel-Aliaga *et al.* 2004) (Figure 1, I–K).

Sequencing of the *Ctr9^12P023^* allele revealed a nonsense mutation early in the open reading frame (Figure 1L). This allele is likely a null, as all phenotypes were similar for *Ctr9^12P023^* homozygotes and hemizygotes (over a *Ctr9* deficiency) (Figure 1K). Early *Ctr9* expression revealed a maternal load, followed by ubiquitous zygotic expression at St4–6. However, from St7 onwards, expression is more restricted, with the strongest signal observed in the CNS (Berkeley Drosophila Genome Project; http://insitu.fruitfly.org). In order to confirm that it is the *Ctr9^12P023^* allele that causes lethality, we generated a *UAS-Ctr9* transgene, and utilized *insc-Gal4* to drive *Ctr9* selectively in the developing nervous system. Hemizygous *Ctr9* mutants (*Ctr9^12P023^*/*Ctr9^Df^*) developed into late embryo stage (AFT), with some hatching into first instar larvae (L1). However, larvae were nonmotile and died. Rescue of *Ctr9* mutants by *insc > Ctr9* completely rescued all Ap cluster phenotypes, including restoration of FMRFa and Nplp1, as well as reducing Ap neuron numbers to wild type (Figure 1, G, H, and K). We also observed increased survival, as the rescued animals produced 20% motile L1 larvae that survived into late L1 stage (*n* = 60 larvae).

We conclude that *Ctr9* is a highly conserved and essential gene in *Drosophila*, and that one important site of its function is the developing nervous system.

### Ctr9 mutants show increased NB and daughter proliferation

The additional Ap neurons observed in *Ctr9* mutants prompted us to investigate the proliferation profile in the NB5-6T lineage. Because of the programmed Type I > 0 switch in NB5-6T, and the stereotyped cell cycle exit of the NB at St15, increased Ap neuron numbers may reflect problems with the Type I > 0 switch and/or NB cell cycle exit.

**Figure 2:**
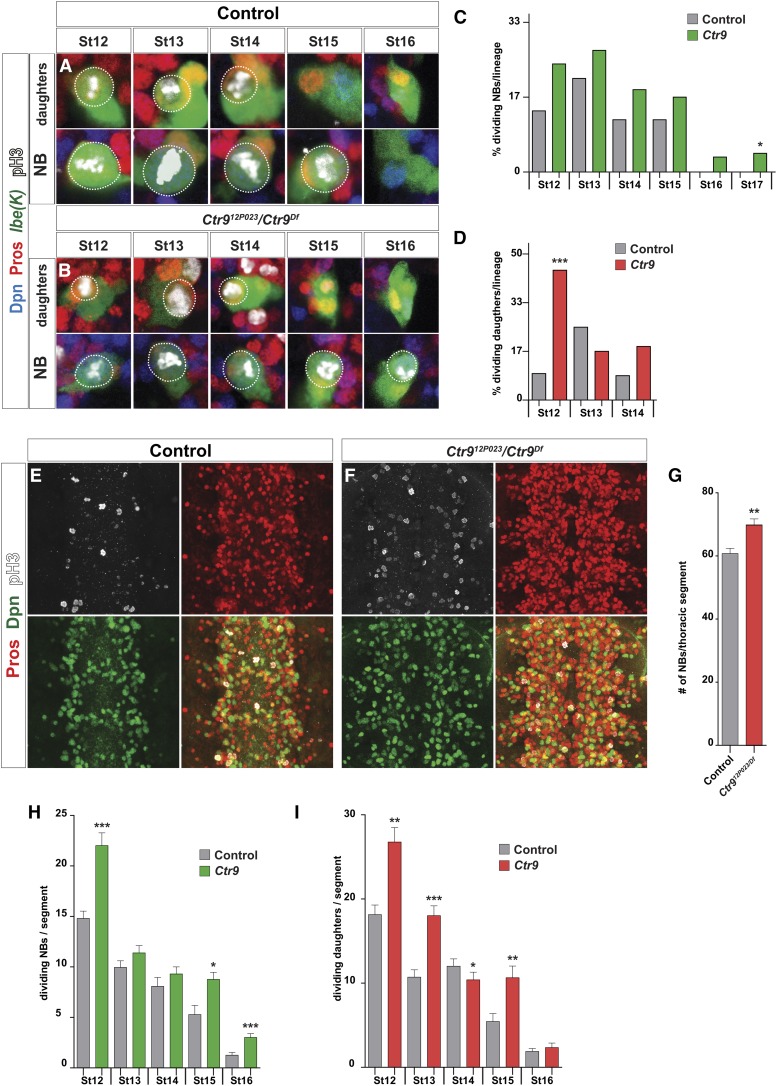
*Ctr9* controls proliferation in the embryonic VNC. (A and B) Dividing NBs and daughters in the NB5-6T lineage can be identified by expression of GFP, Dpn, Pros, and PH3; NBs are GFP+, Dpn+, asymmetric cortical Pros+ and PH3+, while daughters are GFP+, Dpn−, nuclear Pros+ and PH3+. (A) In control, the NB divides until St15 while daughters divide until St14 (dividing cells are circled). (B) In *Ctr9* mutants, NB divisions are observed also at St16 and St17. (A–B): Stain is anti-PH3, anti-Dpn, anti-Pros and anti-GFP. Magnification is 400X. In contrast, daughter divisions are not markedly extended into later stages. (C and D) Quantification of NB and daughter divisions in the NB5-6T lineage (* *P* ≤ 0.05; ** *P* ≤ 0.01; *** *P* ≤ 0.001; *n* ≥ 9 lineages; two-sided Chi-squared test ± SEM) reveals aberrant divisions at St16 and St17 in *Ctr9*. Daughter divisions are elevated at St12, but do not extend aberrantly into St15 and onward. (E and F) Global mitotic analysis in the VNC reveals increased divisions in *Ctr9* mutants. (E–F): Stain is anti-Pros, anti-Dpn and anti-PH3. Magnification is 200X. In addition, the VNC appears enlarged and contains supernumerary Pros and Dpn cells. (G) Quantification of NB numbers (Dpn+/Pros-cytoplasmic) in T2 and T3 segments reveals a minor but significant increase in NB numbers (** *P* ≤ 0.01; Student’s two-tailed *t*-test ± SD; *n* ≥ 20 segments). (H and I) Quantification of NB and daughter divisions in T2 and T3 segments reveals increased NB and daughter divisions at multiple stages (* *P* ≤ 0.05; ** *P* ≤ 0.01; *** *P* ≤ 0.001; Student’s two-tailed *t*-test ± SD; *n* ≥ 14 segments). Genotypes: (A) *lbe*(K)-*EGFP*; (B) *lbe*(K)-*EGFP*, *Ctr9^12P023^/Ctr9^Df^* = *Df(3L)BSC250*; (E) *OregonR*; (F) *Ctr9^12P023^/Ctr9^Df^* = *Df(3L)BSC250*.

To resolve the origin of extra Ap neurons in *Ctr9* mutants, we used anti-Deadpan (Dpn), anti-Prospero (Pros) and anti-PH3, because they can be used to discriminate dividing NBs and daughter cells; NBs express Dpn and have asymmetrically distributed cortical Pros and PH3, whereas daughters are Dpn-negative and have nuclear Pros and PH3 (Figure 2A) (Baumgardt *et al.* 2014). In the wild-type lineage, we observed daughter divisions in NB5-6T up until St14, where the NB divided into St15 (Figure 2, A, C, and D). The presence of dividing daughters at St14, after the Type I > 0 switch occurred at St12, stems from the fact that while NBs divide every 40 min, daughters divide 100 min after they are born, and hence Type I daughters (GMCs) generated earlier in the lineage divide with a delay (Baumgardt *et al.* 2014; Hartenstein *et al.* 1987). In *Ctr9* mutants, we observed a minor increase in daughter divisions at St12, but thereafter no evidence for prolonged daughter divisions (Figure 2D). We observed a stronger effect on NB divisions, with increased prolonged divisions into St16 and St17 (Figure 2, B and C).

To further address the role of *Ctr9* in proliferation control, we analyzed the NB3-3A lineage, which can be readily identified by expression of *eagle* and *eagle*-reporters (Dittrich *et al.* 1997; Lundell and Hirsh 1998). NB3-3A has a short Type I window, followed by a long Type 0 window, with the NB exiting the cell cycle at a defined stage (Supplemental Material, Figure S1D). Hence, it is an informative lineage for identifying daughter switch phenotypes, as well as NB exit problems (Baumgardt *et al.* 2014; Bivik *et al.* 2016). Using the abovementioned markers, we followed NB and daughter proliferation in NB3-3A from St13 to St16. In *Ctr9* mutants we observed an increase in NB divisions at St13, and aberrant extension of NB divisions into St16. In contrast, we did not observe any daughter divisions during these stages in control or *Ctr9* mutants (Figure S1, A–D).

To address if *Ctr9* affects proliferation globally in the VNC, we used the same markers and analyzed the thoracic VNC between St12 and St16. This revealed a significant increase in proliferation in both NBs and daughter cells, albeit to different extents at different stages (Figure 2, E, F, H, and I). *Ctr9* mutant VNCs appeared enlarged and displayed an apparent increase in the number of Pros and Dpn cells (Figure 2, E and F). Indeed, quantification of NBs (Dpn+/Pros-cytoplasmic) revealed a small but significant increase in the number of NBs (Figure 2G).

We conclude that *Ctr9* mutants show an increased number of NBs, and elevated and extended NB divisions, both in the NB5-6T and NB3-3A lineages, as well as globally. There is elevated daughter proliferation globally, but minor daughter proliferation effects in NB5-6T, and no apparent daughter effects in NB3-3A.

### hyrax (Cdc73/Parafibromin/Hrpt2) affects Ap cluster generation

The additional Ap neurons and loss of Nplp1 and FMRFa observed in *Ctr9* mutants prompted us to use this sensitive read-out to investigate the role of other Paf1C members. As previously mentioned, the Paf1C members have not been extensively studied in *Drosophila*, and there is not an abundance of alleles available for these genes. However, strong alleles have been identified for the *hyrax* gene (Cdc73/Parafibromin/Hrpt2) (Mosimann *et al.* 2006). Therefore, we analyzed expression of the Ap cluster markers Eya, Nplp1, and FMRFa in a *hyx^2^*/*hyx^3^* allelic combination. We observed a weak but significant effect on all three markers (Figure S2). These effects mimicked the *Ctr9* mutants, with an increase of Eya (Ap cell numbers) but reduction of Nplp1 and FMRFa (Figure S2). We conclude that at least one additional *Drosophila* Paf1C member phenocopies *Ctr9*.

### Ctr9 affects E2f1, Grh, Antp, and H3K4me3 expression in the embryonic VNC

**Figure 3:**
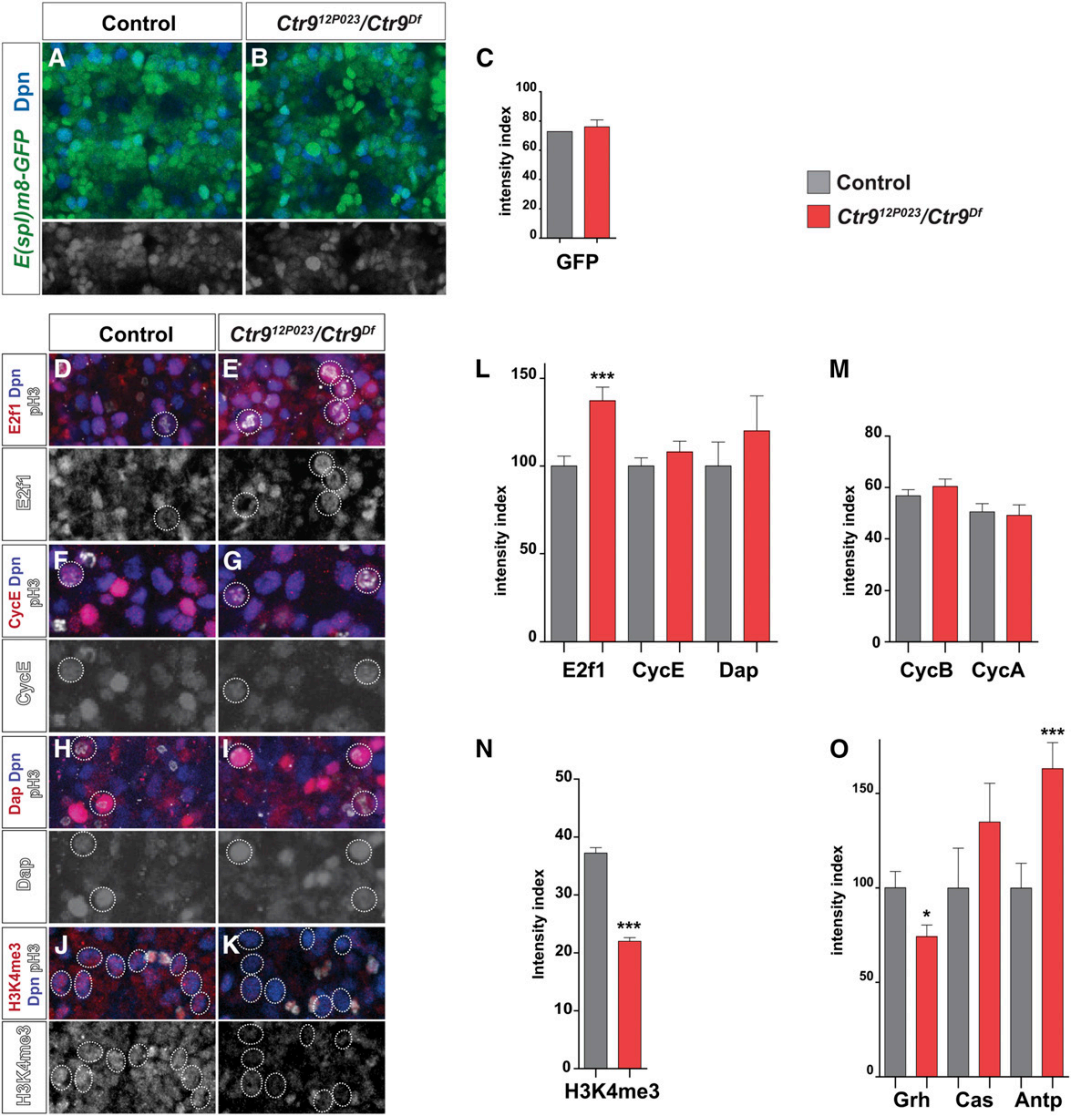
*Ctr9* affects E2f1, Grh, Antp and H3K4me3 expression in the embryonic VNC. (A and B) Expression of *E(spl)-HLHm8-GFP* in control and *Ctr9* mutants, at St14, T2-T3. GFP expression is not apparently affected in *Ctr9* mutants. anti-Dpn and anti-GFP stained. Magnification 400X. (C) Quantification of GFP levels in NBs at St14 reveals no significant effect (Student’s two-tailed *t*-test ± SD; *n* ≥ 127 NBs). (D–K) Expression of E2f1, CycE, Dap, and H3K4me3 in control and *Ctr9* mutants, in thoracic T2–T3 segments, at St14. Dividing NBs are circled. Levels of E2f1 are increased in dividing NBs, while H3K4me3 is decreased. (L–O) Quantification of Dap, CycE, E2f1, CycA, CycB, H3K4me3, Grh, Cas, and Antp expression levels in dividing NBs, in control and *Ctr9* mutants, in T2 and T3 segments, at St14. Expression of E2f1 is significantly increased in *Ctr9* mutants, while the other four cell cycle proteins are not affected. (D–K) anti-Dpn, anti-PH3, anti-E2f1 stained (D–E), anti-CycE (F–G), anti-Dap (H–I) and anti-H3K27me3 (J–K). Magnification if 400X. With regard to the developmental regulators, *Ctr9* mutants display elevated Antp expression and lowered Grh expression, while Cas was not affected. H3K4me3 is reduced in *Ctr9* mutants (* *P* ≤ 0.05; *** *P* ≤ 0.001; Student’s two-tailed *t*-test ± SD; *n* ≥ 85 NBs).

Previous studies have linked Paf1C components to the Notch pathway, exemplified by its involvement in the regulation of Notch downstream genes, such as the HES genes (Akanuma *et al.* 2007; Tenney *et al.* 2006). Given that Notch signaling is known to affect both the NB selection process and the Type I > 0 daughter proliferation switch in the VNC (Lehmann *et al.* 1983; Poulson 1937; Ulvklo *et al.* 2012), we addressed the possible connection between *Ctr9* and Notch signaling. To this end, we analyzed expression of the Notch target gene *E(spl)-HLHm8*, as evidenced by expression of an *E(spl)-HLHm8-GFP* reporter transgene that reports on Notch signaling in developing NBs (Ulvklo *et al.* 2012). However, this revealed no obvious effect on GFP expression in NBs or daughter cells, at St14 (Figure 3, A–C).

The global proliferation phenotypes we observed in *Ctr9* mutants prompted us to also address expression of several key regulators involved in NB and daughter proliferation control in the VNC. We focused on the two temporal factors, Castor (Cas) and Grainy head (Grh), as well as the Hox factor Antennapedia (Antp), again focusing on dividing NBs in the T2–T3 segments (Baumgardt *et al.* 2014). These studies revealed no effect on Cas and a partial loss of Grh, while Antp was upregulated (Figure 3O).

A number of cell cycle genes have been identified as key components for NB and daughter proliferation control in the *Drosophila* embryonic VNC (Baumgardt *et al.* 2014). To understand the underlying mechanisms behind the increased proliferation in *Ctr9* mutants, we addressed the expression of Cyclin A (CycA), Cyclin B (CycB), Cyclin E (CycE), E2f1, and Dacapo (Dap). Because both the E2f1 and Dap protein levels cycle during the cell cycle, with nondetectable levels during S-phase (Baumgardt *et al.* 2014), we quantified dividing NBs, focusing on thoracic segments T2–T3, at St14. We noted a significant increase in E2f1 expression in *Ctr9* mutants, while the other four cell cycle proteins were not affected (Figure 3, D–I, L, and M).

Finally, given the known role of Paf1C in histone modifications, we addressed the level of H3K4me3 modification, using an antibody directed against this modification, in NBs at St14, again focusing on thoracic segments T2–T3. We noted an apparent overall decrease in H3K4me3 in the VNC (Figure 3, J and K), and quantification revealed significant reduction in the expression levels in NBs (Figure 3N).

### Ctr9 regulates a number of neuropeptides

**Figure 4:**
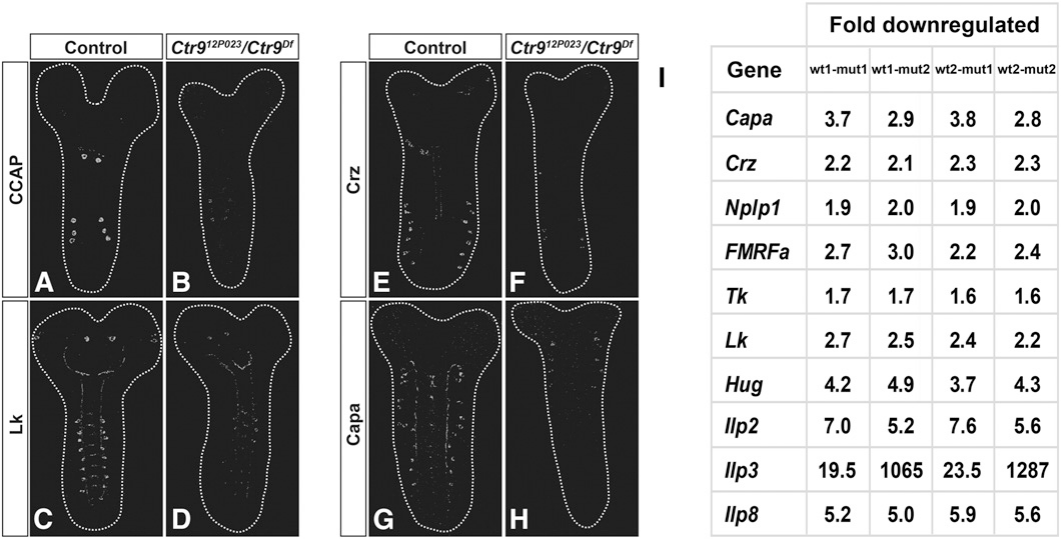
*Ctr9* affects expression of several neuropeptide genes. (A–H) Expression of CCAP, Crz, Lk, and Capa neuropeptides in the CNS, at AFT stage, in control and *Ctr9* mutants. For each neuropeptide, CNSs from control and mutant were stained on the same slide and scanned using identical confocal settings. Expression of all four neuropeptides is affected in *Ctr9* mutants, with levels down in all expressing cells and expression beyond detection for many cells. (A–B) Anti-CCAP stained (C–D) Lk. stained (E–F) Crz Stained. (G–H) Capa Stained. Magnification is 200x. (I) Transcriptome analysis (RNA sequencing) of control and *Ctr9* mutants, from RNA prepared at stage AFT; pair-wise comparison of two independent wild-type and mutant samples. In line with the antibody analysis, transcriptome analysis reveals downregulation of *FMRFa*, *Nplp1*, *Crz*, *Lk*, and *Capa* neuropeptide genes. In addition, *Tachykinin*, *Hugin*, and the *Ilp2*, *Ilp3*, and *Ilp8* genes were also downregulated. *Ccap* was not downregulated.

The near complete loss of the FMRFa and Nplp1 neuropeptides prompted us to analyze expression of a number of other neuropeptides. To this end, we generated antibodies to four neuropeptides known to be expressed in the developing CNS (see *Materials and Methods*): Crustacean cardioactive peptide (CCAP), Leucokinin (Lk), Corazonin (Crz), and Capability (Capa) (Park *et al.* 2008). Analyzing late-stage embryos, we noted strong reduction in the expression of all four neuropeptides in *Ctr9* mutants, both with respect to intensity of expression and apparent loss of expression in some cells (Figure 4, A–H).

**Figure 5:**
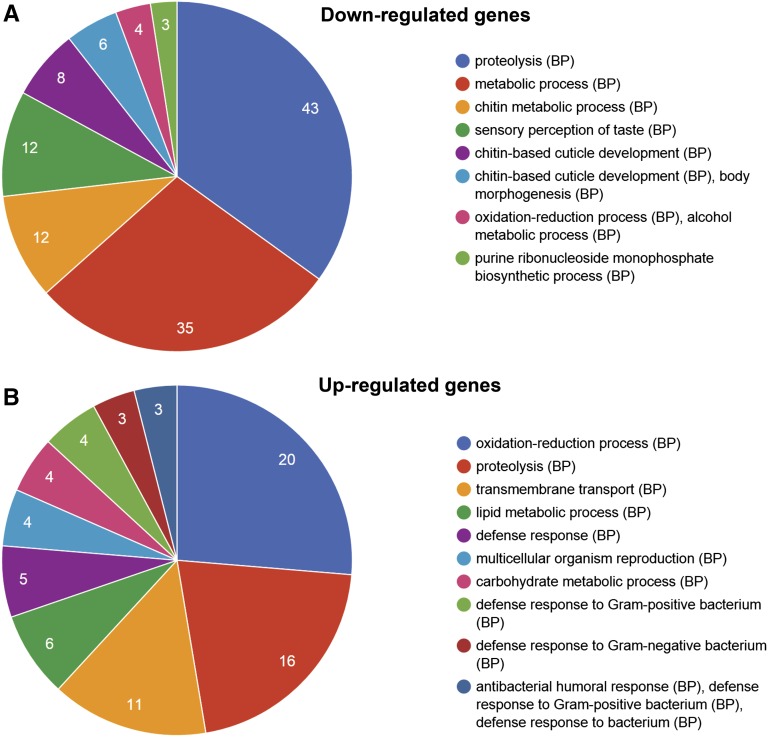
*Ctr9* affects expression of metabolic genes. Pair-wise comparison between two control and two *Ctr9* mutant RNA samples, all isolated at stage AFT, reveals a significant enrichment for gene expression changes in genes involved in metabolism. (A) Down-regulated genes. (B) Up-regulated genes. BP, Biological Process.

To validate these findings, and to obtain a broader picture of the gene expression changes in *Ctr9* mutants, we analyzed the transcriptome in late-stage embryos. To this end, two independent RNA preparations were extracted from wild-type and *Ctr9* mutant embryos at late embryonic stage (AFT). The four RNA samples was subjected to sequencing on the Illumina HiSeq2500, with 50 bp single reads, at an average depth of 38M reads per sample. The four pair-wise comparisons between the two wild-type and two *Ctr9* mutant biological replicates were in line with the antibody staining, and revealed downregulation of five out of the six neuropeptide genes tested (Figure 4I). We also noted downregulation of the *Tachykinin* (*Tk*) and *Hugin* (*Hug*) neuropeptide genes, as well as three of the *Insulin-like peptide* genes (*Ilp2*, *Ilp3*, and *Ilp8*) (Figure 5I).

Hence, in addition to FMRFa and Nplp1, a number of other terminal differentiation genes (such as neuropeptides) are affected in *Ctr9* mutants.

### Ctr9 mutants display changes in gene expression of homeostatic genes

Pair-wise comparisons between the two wild-type and two *Ctr9* mutant transcriptomes revealed that 252 genes were ≥2-fold upregulated in *Ctr9* mutants, while 797 genes were ≥2-fold downregulated (Table S1). The bias toward gene downregulation is in agreement with the important role of the Paf1 complex in Pol II transcription. In line with the involvement of Paf1C in the regulation of Notch downstream genes, such as the HES genes (Akanuma *et al.* 2007; Tenney *et al.* 2006), we noted downregulation of six previously identified Notch target genes (Krejci *et al.* 2009). These include three other *E(spl)* complex genes: *E(spl)m4*, *E(spl)m5-HLH*, *E(spl)m6*, *Peb*, *Him*, and *stg* (Table S1). Looking at the GO profile of the misregulated genes, we noted a significant overrepresentation of genes involved in adult homeostatic processes, such as proteolysis, metabolic, and oxidative processes (Figure 5, A and B).

## Discussion

The Paf1C is involved in a number of transcriptional and epigenetic events. Here, we provide the first detailed study of the *Drosophila* gene encoding one of the central components, *Ctr9*, and find that this gene is essential in *Drosophila*. Our results point to *Ctr9* contributing to NB selection, proliferation control, terminal differentiation, and control of metabolic gene expression. Analysis of histone modifications further reveals a clear reduction in H3K4me3, in line with the known role of Paf1C in controlling this modification. We also observed a phenocopy of the *Ctr9* effects on the Ap cluster neurons, albeit milder, in *hyrax* mutants (Cdc73/Parafibromin/Hrpt2). This lends some support to the notion that the effects of *Ctr9* may occur during *Drosophila* embryogenesis, within the context of a canonical Paf1C.

### Involvement of Ctr9 in proliferation control

We observed elevated and extended NB divisions in *Ctr9* mutants, both globally and in two specific lineages. We also noted a minor increase in overall NB numbers. These extra NBs may stem from defects in the lateral inhibition process and/or from dedifferentiation of daughter cells into NBs. Irrespective of their origin(s), the extra NBs observed in *Ctr9* raises the question of whether proliferation effects are solely due to the presence of supernumerary NBs. However, we do not favor this interpretation because global NB proliferation was robustly elevated and extended during several stages while the increase in NB numbers was minimal. In addition, we did not observe any extra NB5-6T or NB3-3A NBs, and we still observed elevated and extended NB proliferation in these single lineages.

Regarding increased daughter proliferation, we noted minor effects in NB5-6T but no effects in NB3-3A. Globally, we did observe elevated daughter divisions, but these may stem from elevated and extended NB divisions. Hence, we favor the interpretation that increased daughter proliferation in *Ctr9* mutants primarily results from a combination of supernumerary NBs and extended NB proliferation, with the latter being the main driver. However, we cannot rule out problems with the precise execution of the Type I > 0 daughter proliferation switch.

What are the underlying gene expression changes driving increased proliferation in *Ctr9* mutants? While we noted increase in E2f1 expression, E2f1 only plays a minor role in VNC proliferation (Baumgardt *et al.* 2014). In mammals, Parafibromin (Cdc73/Hyrax) and Paf1 were found to control expression of a number of cyclins (Moniaux *et al.* 2009; Woodard *et al.* 2005). However, we did not observe any effects on CycE, CycA, or CycB expression. We did not analyze CycD expression, but it was previously found not to be a key regulator of embryonic VNC proliferation (Baumgardt *et al.* 2014). In addition to E2f1 upregulation, we noted weak expression changes of two transcription factors known to be important for VNC proliferation control (Baumgardt *et al.* 2014): downregulation of Grh and upregulation of Antp. Both Grh and Antp were found to repress E2f1 (Baumgardt *et al.* 2014). Hence the downregulation of Grh in *Ctr9* could, at least in part, explain the upregulation of E2f1, while the upregulation of Antp does not fit easily with its role in repressing E2f1 expression.

### Paf1C/Ctr9 is important for proper Notch signaling

We noted supernumerary NBs in *Ctr9* mutants, and since it is well known that NB selection during early VNC formation critically depends upon Notch signaling (Lehmann *et al.* 1983; Poulson 1937), it is tempting to speculate that these effects are due to affects upon early Notch signaling. This notion is further supported by other studies that have revealed a link between Paf1C and Notch signaling in both *Drosophila* and zebrafish (Akanuma *et al.* 2007; Tenney *et al.* 2006). Moreover, Paf1C is essential in recruiting the Rad6-Bre1 H2B ubiquitin ligase complex (Tomson and Arndt 2013), and Bre1 has been found have an important effect on Notch signaling, evidenced in part by downregulation of the *E(spl)-HLH* (HES) Notch target genes (Bray *et al.* 2005). Surprisingly, we did not find any apparent effects upon *E(spl)m8-GFP* expression at St14. However, in the transcriptome analysis we did note downregulation of six previously identified Notch target genes (Krejci *et al.* 2009), including three other *E(spl)* complex genes: *E(spl)m4*, *E(spl)m5-HLH*, *E(spl)m6*, *Peb*, *Him*, and *stg* (Table S1). In addition to the possible connection between *Ctr9*, Paf1C, and Notch signaling with respect to NB selection, our studies have previously identified that late Notch signaling also controls the Type I > 0 daughter proliferation switch in the developing VNC (Bivik *et al.* 2016; Ulvklo *et al.* 2012). Hence, we propose that in several of the Ctr9 mutant phenotypes, extra NBs and increased daughter proliferation are likely due to a connection between *Ctr9* and the Notch pathway. Against the backdrop of growing evidence for a tight interplay between Paf1C/Ctr9 and Notch signaling, as well as cell cycle gene expression, it is not surprising that Paf1C/Ctr9 is emerging as an important factor in human cancer (Dey *et al.* 2011; Hanks *et al.* 2014; Muntean *et al.* 2010; Newey *et al.* 2009; Takahashi *et al.* 2011; Zeng and Xu 2015).

### Ctr9 and the Paf1C: connections to early embryonic development

A number of studies have identified important roles for Paf1C in early mouse development, as well as in embryonic stem cell identity, and *Ctr9* knockdown and Parafibromin (*Hrpt2*) knockout completely blocks early mouse embryo development (Ding *et al.* 2009; Ponnusamy *et al.* 2009; Wang *et al.* 2008; Zhang *et al.* 2013).

In contrast to these effects, we find rather mild effects of *Ctr9* mutations on embryonic development in *Drosophila*, and although the gene is essential, embryos develop until completion, with a small number even hatching out as larvae (although they immediately die). The reason for this embryonic “escape” from *Ctr9* function may be due to the maternal loading of the genes, and hence embryonic development can rely solely upon maternal Ctr9 RNA and protein.

### Ctr9 regulates metabolism genes

The Paf1C has previously been linked to dynamic gene regulation, being involved in heat-shock response genes in *Drosophila* (Hsp70) (Adelman *et al.* 2006), and in the regulation of metabolic genes, *i.e.*, those involved in lipid and nucleic acid metabolism in yeast (Betz *et al.* 2002). In line with these findings, our genome-wide transcriptome analysis revealed that *Ctr9* mutants were enriched for expression changes in a number of metabolic genes, including lipid metabolism and nucleotide biosynthesis (Figure 5 and Table S1). It would thus appear that the connection between Paf1C and metabolic gene regulation is conserved in eukaryotes.

## 

## Supplementary Material

Supplemental Material
