# Supplementary material for: Ctr9, a Key Component of the Paf1 Complex, Affects Proliferation and Terminal Differentiation in the Developing Drosophila Nervous System
Source: G3 (Bethesda). 2016 Aug 11;6(10):3229–39. doi: 10.1534/g3.116.034231 (PMC5068944; doi:10.1534/g3.116.034231)
Supplement: Supplemental Material [file supp_6_10_3229__index.html]

Ctr9, a Key Component of the Paf1 Complex, Affects Proliferation and Terminal Differentiation in the Developing Drosophila Nervous System — Supplemental Material 

# Ctr9, a Key Component of the Paf1 Complex, Affects Proliferation and Terminal Differentiation in the Developing *Drosophila* Nervous System

## Supplemental Material for Bahrampour and Thor, 2016

**Files in this Data Supplement:**

- Table S1 - Genes two-fold up- or down-regulated in *Ctr9* mutant embryos. (.xlsx, 128 KB)
- Figure S1 - *Ctr9* controls proliferation in NB3-3A. (.ai, 1.17 MB)
- Figure S2 - *hyrax* affects Ap cluster generation. (.ai, 1.13 MB)
