# Supplementary figures and images for: Ctr9, a Key Component of the Paf1 Complex, Affects Proliferation and Terminal Differentiation in the Developing Drosophila Nervous System
Source: G3 (Bethesda). 2016 Aug 11;6(10):3229–39. doi: 10.1534/g3.116.034231 (PMC5068944; doi:10.1534/g3.116.034231)

# Supplemental Figure 1

## *Ctr9* Controls NB Proliferation in NB3-3A

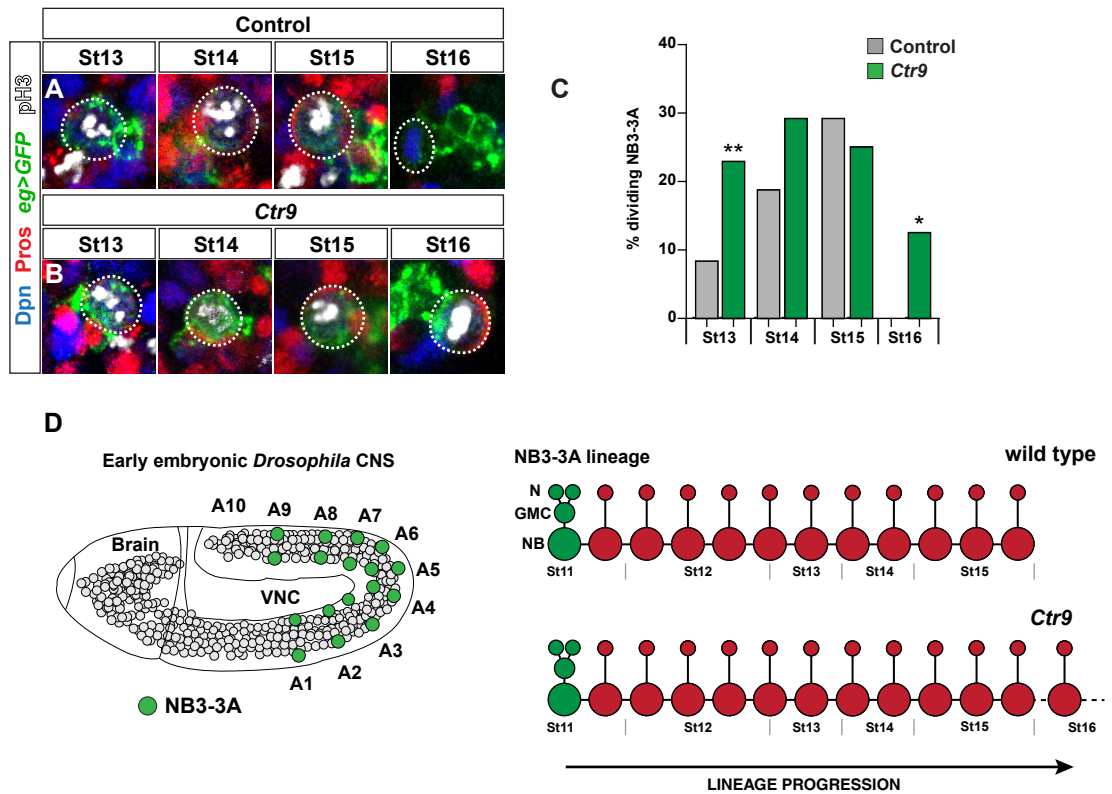

Supplement: Supplemental Material [file supp_g3.116.034231_FigureS1.ps]

## Supplemental Figure 2

### *hyrax* affects Ap cluster generation

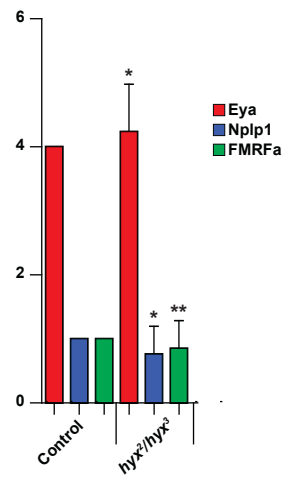

Supplement: Supplemental Material [file supp_g3.116.034231_FigureS2.ps]
